# Minimal residual disease in *EGFR*-mutant non-small-cell lung cancer

**DOI:** 10.3389/fonc.2022.1002714

**Published:** 2022-09-21

**Authors:** Nathan T. Bain, Yang Wang, Surein Arulananda

**Affiliations:** ^1^Department of Medical Oncology, Monash Health, Clayton, VIC, Australia; ^2^School of Clinical Sciences, Faculty of Medicine, Monash University, Clayton, VIC, Australia; ^3^Centre for Cancer Research, Hudson Institute of Medical Research, Clayton, VIC, Australia; ^4^Department of Medical Oncology, Dana-Farber Cancer Institute, Boston, MA, United States

**Keywords:** Non-small-cell lung cancer, minimal residual disease, EGFR, apoptosis, senescence

## Abstract

Targeted therapy with epidermal growth factor receptor (EGFR) tyrosine kinase inhibitors (TKIs) is an effective treatment for *EGFR-*mutant non-small-cell lung cancer (NSCLC), however most patients invariably relapse after a period of minimal residual disease (MRD). This mini-review explores the mechanistic pathways leading to tumour dormancy, cellular senescence and epigenetic changes involving YAP/TEAD activation. We describe the various approaches of utilising TKIs in combination with agents to intensify initial depth of response, enhance apoptosis and target senescence-like dormancy. This mini-review will also highlight the potential novel therapies under development targeting MRD to improve outcomes for patients with *EGFR*-mutant NSCLC.

## Introduction

Lung cancer, the second most commonly diagnosed cancer in 2020, remains the leading cause of global cancer death with an estimated 1.8 million deaths worldwide (1). Early-stage non-small-cell lung cancer (NSCLC) is managed by curative intent treatments such as surgical resection or ablative radiotherapy (2). Depending on histological and nodal staging, this is followed in most cases by a course of adjuvant systemic chemotherapy and/or immunotherapy to reduce the risk of cancer recurrence (3). Following complete surgical resection, the additional survival benefit conferred by platinum-based adjuvant chemotherapy increases with pathological stage (4, 5). Nevertheless, recurrence rates remain high with 30% to 55% of patients with NSCLC developing recurrence (6). This is largely attributed to a propensity for NSCLC to persist within the body following curative and adjuvant treatments in the form of minimal residual disease (MRD): micrometastatic deposits or circulating tumour cells (7, 8). Detecting MRD has become standard in some haematological malignancies, and liquid biopsy for circulating tumour DNA (ctDNA) is an active area of interest in several solid tumour types (9). However, the biology driving MRD in solid cancers seems to be highly variable between solid tumour types, with some clinically important differences recently associated with the presence of oncogenic driver mutations.

Oncogenic driver mutations have emerged as key therapeutic targets. EGFR is a receptor tyrosine kinase involved in extracellular growth factor signalling which is associated with growth, proliferation and survival (10). EGFR mutations are common and are seen in 10-15% of North American/European NSCLC, but up to 30-50% of East Asian patients.(ref) They are characteristically seen in pulmonary adenocarcinoma with bronchoalveolar features, particularly in patients without a history of smoking. *EGFR* mutations such as *exon 19 deletion* and *L858R* substitution lead to dysregulated EGFR activation and anti-apoptotic signalling can be achieved by mutation within the four exons (18-21) encoding the part of the tyrosine kinase domain clustered around the ATP-binding pocket of the enzyme.

Like other oncogenic mutations, *EGFR* mutations display “clonal” behaviour *in vivo*: they arise within individual cells which propagate and diverge into different lineages which undergo a quasi-Darwinian process where tumour cell populations descended from a progenitor or ‘clone” acquire successive somatic mutations that confer a selective advantage (11). This inevitably results in solid tumours which display highly heterologous cell populations. Exposure to systemic anti-cancer treatments has the effect of a selective pressure selecting for cancer cell lineages which contain or develop treatment resistance. As treatment resistance is only present in a small proportion of treatment-naïve cancer cells, initial clinical response can often be quite promising. Unfortunately, these responses lack durability, either over time or in the absence of ongoing systemic treatment. An archetypal example of this pattern can be seen in the response of *EGFR-*mutant NSCLC to tyrosine kinase inhibitors.

In advanced *EGFR-*mutant NSCLC, gefitinib (12), and erlotinib (13) were the first TKIs to demonstrate improved progression free survival (PFS) in comparison with platinum doublet chemotherapy. Osimertinib, a third generation TKI with improved binding activity, demonstrated high response rates as well as superior PFS compared to gefitinib and erlotinib in the advanced disease setting, and is the current standard-of-care therapy (14). Interestingly, while long term responders were observed in both the osimertinib and gefitinib/erlotinib groups, the survival rates for the FLAURA study at 3 years was 54% in the osimertinib group (15). Unfortunately, despite good initial response to treatment, TKIs do not in most patients create a durable response. This pattern of failure suggests that the effect of TKIs on *EGFR-*mutant NSCLC is more likely to be suppression rather than eradication. This distinction between cancer suppression and cancer eradication, calls into question the true value of TKIs in the adjuvant setting where the elimination (not suppression) of MRD is the therapeutic goal.

In the setting of completely resected *EGFR-*mutant NSCLC, gefitinib demonstrated superior PFS compared to standard platinum doublet adjuvant chemotherapy (16). However, subsequent follow-up showed that this initial response did not translate into an improvement in overall survival (17). The landmark ADAURA study (18) comparing a three-year course of adjuvant osimertinib versus placebo in completely resected stage IB to IIIA *EGFR-*mutant NSCLC demonstrated an early advantage in disease free survival of 89% vs 52% at 24 months with an overall hazard ratio (HR) 0.2 (99% CI, 0.14 to 0.30). Despite this significant difference, questions have been raised about clinical significance of disease-free survival as an endpoint, as well as the variable use of adjuvant chemotherapy between treatment groups (19). Overall survival data is eagerly awaited. It may well be that the effect of TKI treatment in the adjuvant setting turns out to be one of MRD-suppression rather than MRD-eradication.

It is important to bear in mind that the persistence of MRD to TKI treatment is distinct from bona fide treatment resistance which is classically characterised as the development or presence of specific mutations such as *T790M EGFR* mutations. These persister cells on the other hand do not by necessity harbour such mutations and are able to maintain viability throughout TKI treatment through other, less well understood mechanisms. It has been observed that *EGFR-*mutant tumour cells can enter a drug-tolerant state reminiscent of cellular senescence that enables ongoing survival predominantly through resistance to or inhibition of apoptosis (20).

## Apoptosis and senescence in *EGFR*-mutant cancer cells

Apoptosis is an inducible, stepwise process of programmed cell death which can be classified into two broad pathways. The extrinsic pathway which is initiated by a class of cell membrane proteins known as death receptors, and the intrinsic pathway which is initiated through elaborate intracellular processes which invariably converge at the mitochondrial outer membrane. This leads to permeabilization and the subsequent release of cytochrome c into the cytoplasm. The release of cytochrome c from the mitochondria is stimulated by pro-apoptotic members of the BCL-2 family (i.e., BAX, BAK, BIM, BMF, BID and BAD) and inhibited by pro-survival members of the same family, such as BCL-2, BCL- XL and MCL1. Importantly, the efficacy of TKIs in *EGFR-*mutant NSCLC is reliant upon their ability to induce apoptosis by modulating the expression of members of the BCL-2 family (21). The pro-apoptotic protein BIM is phosphorylated by several cell survival pathways including ERK1/2 and MAPK1. The knockdown of *BIM* by small interfering RNA was observed to attenuate apoptosis induced by EGFR TKIs in cancer cell lines *in vitro* (22). Patients with *EGFR-*mutant NSCLC harbouring a *BIM* deletion polymorphism exhibit greater resistance to TKI treatment (23). An important pre-clinical study showed that a primary means by which cancer cells may evade apoptosis both *in vitro* and *in vivo* is by entering into a state of dormancy or senescence (24).

Cellular senescence is broadly defined as a viable, non-proliferative state akin to cellular dormancy. Replicative senescence is attributed to the progressive loss of protective telomeric DNA with mitotic cellular division and is mediated predominantly through the actions of p53 (25). Inducible senescence is a more varied phenomenon and can result from exposure to noxious and/or oncogenic stimuli such as ionising radiation or oxidative stress. One unique form of inducible senescence is *oncogene-induced* cellular *senescence* (OIS*)*, which was initially demonstrated *in vitro* by the transfection of oncogenic *HRAS V12* into murine fibroblasts which produced a strong anti-proliferative effect associated with activation of *p16^INK4A^* and *p19^ARF^* (26). The cell signalling pathways mediating OIS are complex but both *pRB* and *p53* are involved in maintaining proliferative arrest (27). Rather than being maladaptive, senescence in this context is thought to be anti-oncogenic. Upregulation of oncogenes such as *RAS* and *RAF* have been observed to induce senescence in several *in vitro* models (28). However, senescence may also be playing an anti-therapeutic role in the context of anti-cancer therapies. TKIs such as gefitinib have also been observed to induce cellular senescence in malignant cells both *in vitro* and *ex vivo* (29). The escape/evasion of EGFR inhibition poses a hard problem to current TKI treatments targeting *EGFR-*mutant NSCLC.

## Therapeutic strategies to enhance Osimertinib response

### Targeting senescence-like cell dormancy

A novel area of significant interest is targeting cancer cell dormancy itself (24). An *EGFR-*mutant lung cancer cell line exposed to EGFR/MEK inhibition *in vitro* through a combination of osimertinib and trametinib (via a DMSO-containing solution of 100 nM and 30 nM of the former and latter agents respectively) induced a widespread apoptotic response. A small proportion of cells were able to persist throughout this die-off, independent of ERK signalling, by entering a stable dormant state and were still detectable after fifteen weeks of treatment. After drug washout, these dormant cells were observed to proliferate and recolonize the wells within a matter of days. This reversible dormant state shared several characteristics in common with cellular senescence including senescence-associated beta-galactosidase staining, flattened morphology typical of senescent cells, and H3K9Me3-positive nuclear foci.

**Figure 1:**
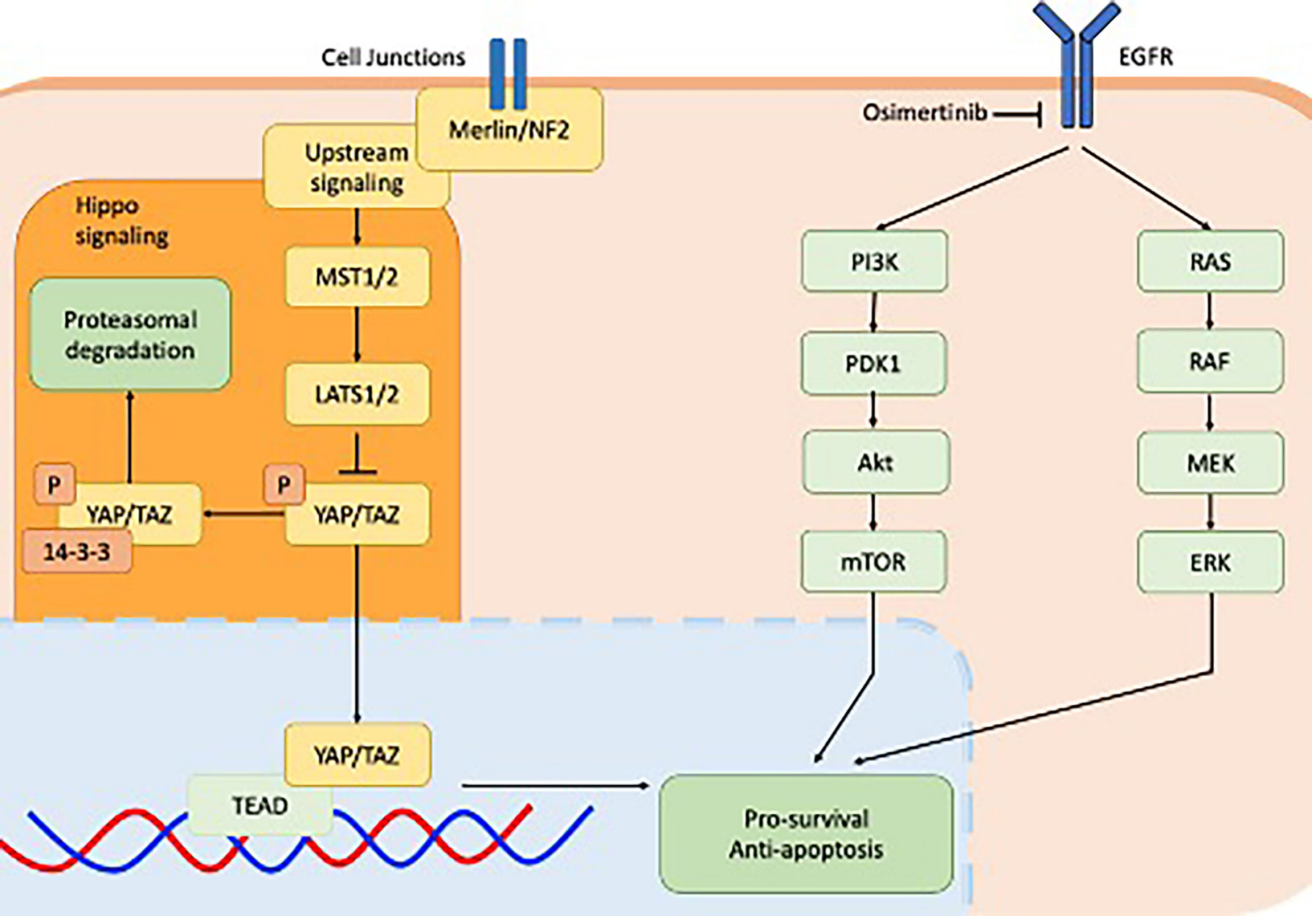
Cell signalling pathways implicated in *EGFR*-mutant non-small-cell lung cancer including downstream signalling of EGFR receptor *via* the PI3K-AKT pathway (green) and MAPK pathway (green) and the YAP/TEAD hippo signalling pathway downstream of Merlin/NF2. Both pathways converge on the apoptosis pathway.

The establishment and maintenance of dormancy was found to be closely associated with YAP/TEAD/Hippo activity as shown in **Figure 1**. Dormant *EGFR-*mutant NSCLC cells expressed significant enrichment of *YAP/TEAD* gene expression signature, which was identified using RNA sequencing. Increased YAP activity and decreased ERK1/2 activity was observed in a patient-derived xenograft *in vivo* model from tumour tissue sampled from a patient with *EGFR-*mutant NSCLC who had undergone partial response following treatment with osimertinib and selumetinib (24). This study went on to demonstrate that co-treatment of *EGFR*-mutant NSCLC cell lines with osimertinib plus XAV939, a tankyrase inhibitor that indirectly inhibits YAP signalling, reduced both the abundance of dormant cells as well as the regrowth of cells following washout. In three separate *YAP1* knockout *EGFR-*mutant NSCLC cell lines, cell dormancy following osimertinib exposure was completely abolished. Co-targeting cell dormancy in combination with EGFR/MEK inhibition as a means of bypassing apoptosis evasion is a promising direction for future therapeutics. This could be accomplished in several ways. For instance, YAP inhibition may be accomplished by direct inhibitors such as MFF, or indirect inhibitors such as the tankyrase inhibitor XAV939. As well, the most effective timing of YAP inhibition will need to be established. YAP inhibitors used upfront in conjunction with EGFR/MEK inhibitors may exhibit different efficacy or toxicity compared to treating with EGFR/MEK inhibitors first and adding YAP inhibitors later when dormancy has been established.

### Targeting apoptosis

If TKI escape/evasion is occurring through the attenuation of the upstream, pro-apoptotic proteins such as BMF and BIM, then direct co-stimulation of apoptotic effectors may potentiate TKI treatments. Venetoclax is a selective and potent inhibitor of BCL-2 that binds to the BH3 domain of BCL-2 and disrupts its ability to interact with the pro-apoptotic protein BIM, thereby inducing apoptosis (30). As a monotherapy, venetoclax has been found to inhibit the growth of high BCL-2-expressing small cell lung cancer *in vitro* (30). Similar pre-clinical work has shown BH3-mimetics targeting BCL-XL and BCL-2 are also effective agents against pleural mesothelioma (31). Perhaps most interesting, the combination of pro-apoptotic BH3-mimetics with additional anti-cancer agents has been observed to produce synergistic anti-tumour effects in several studies. Venetoclax combined with osimertinib significantly enhances apoptosis in *EGFR*-mutant NSCLC cells with acquired osimertinib resistance (32). As well, a strong anti-tumour effect was induced through the combination of ABT-737, a BH3 mimetic which displaces the anti-apoptotic protein BCL-XL, and cisplatin, was observed in a murine NSCLC model (33). Similarly, navitoclax, a BCL-2 inhibitor, was found to work synergistically with 5-fluorouracil in oesophageal cancer cells *in vitro* (34). Potentiating apoptosis may be a reliable way of amplifying or even restoring the efficacy of several anti-cancer regiments.

## Trials

### Intensifying TKI regimens

Various approaches are currently underway to address MRD by combining EGFR TKIs with other therapeutic agents. One such strategy is combining EGFR TKIs with platinum-pemetrexed chemotherapy. The FLAURA2 study is currently underway and attempts to answer this question of whether osimertinib used in combination with chemotherapy would deepen MRD in the first line setting and delay the development of resistance (NCT04035486). The TAKUMI trial randomised sixty-two patients who had developed *T790M* resistance mutation after first line EGFR-TKI therapy to either osimertinib with combination carboplatin-pemetrexed versus osimertinib alone and found that there was no significance difference in median PFS (35). In regards to safety, a meta-analysis of combination chemotherapy with first-generation TKIs found overall increased toxicities most notably myelosuppression and gastrointestinal side effects (36). As well, no specific pattern of toxicity leading to dose modification or discontinuation was observed, although one patient discontinued study treatments due to pneumonitis (37).

Co-targeting EGFR and MEK inhibition is a promising area of research interest given pre-clinical trials have shown efficacy compared to single agent alone. Osimertinib combined with a MEK or ERK inhibitor enhanced apoptosis and prevented the emergence of osimertinib resistance *in vitro* by enhancing osimertinib-induced apoptosis, the prevention of ERK1/2 reactivation, and inhibited the emergence of resistance in osimertinib-sensitive models known to acquire resistance *via* both *T790M*-dependent and *T790M*-independent mechanisms (38, 39). The clinical application of this has yielded mixed results. There have been case studies showing combination osimertinib and MEK inhibitor trametinib have led to an increased response after progression with osimertinib (40, 41), whilst in another study the sequential addition of a MEK inhibitor trametinib in a patient population already pre-treated with prior TKI therapy did not demonstrate efficacy highlighting the importance of patient selection (42). The findings of a phase II study evaluating the combination of osimertinib with MEK 1/2 inhibitor selumetinib will undoubtably be informative as to the *in vivo* efficacy of this combination (NCT03392246).

The strategy of TKI-immunotherapy combinations has been largely limited by the lack of data on the clear benefit and the high rates of toxicities namely interstitial lung disease. Combination osimertinib and durvalumab was associated with a high incidence (38%) of interstitial lung disease (43), and in the TATTON study; the same combination was shown to cause significant toxicities with grade 3 or higher adverse events reported in almost half of patients and interstitial lung disease reported in 22% leading to early discontinuation (44).

## Future perspectives

The current landscape of trials in the *EGFR-*mutant NSCLC space primarily involves osimertinib in combination with agents active against downstream pathways and acquired resistance mechanisms as shown in **Table 1**. While fourth generation EGFR-TKIs are in development to target the most commonly seen emergent mutations *C797S* and *C797X* after third line EGFR-TKI treatment, the authors speculate that these approaches are reactionary and invariably other resistance mechanisms will develop (NCT04862780), (NCT05153408), (NCT05394831). Aurora kinase A activation, another acquired resistance mechanism is shown to play a role in drug resistance to EGFR-TKI treatment and is also the subject of early phase trials involving the addition of Alisertib (NCT04085315) (NCT04479306) (NCT05017025). Other early phase trials are evaluating inhibition of the downstream signalling pathway PI3K/mTOR with sapanisertib (NCT04479306) and MET inhibition with bispecific antibody Amivantamab (NCT02609776) (NCT04965090).

**Table 1:** Currently recruiting *EGFR* mutation non-small-cell lung cancer trials.

Agent	Study	Phase	Target
Amivantamab	NCT02609776 (CHRYSALIS)	Phase I	Human bispecific EGFR-cMET antibody
Osimertinib + Nectiumumab	NCT02496663	Phase I	Human IgG1 monoclonal antibody
Osimertinib + Selumetinib	NCT03392246	Phase II	MEK1/2 inhibitor
Dacomitinib	NCT03755102	Phase I	EGFR inhibitor
Osimertinib + Dacomitinib	NCT03810807	Phase I	EGFR inhibitor
Osimertinib + Telaglenastat	NCT03831932	Phase I	Glutaminase inhibitor
Osimertinib + Telaglenastat	NCT03831932	Phase Ib	Glutaminase inhibitor
Osimertinib + Alisertib	NCT04085315	Phase I	Aurora Kinase A inhibitor
Osimertinib+ Alisertib + Sapanisertib	NCT04479306	Phase Ib	Aurora Kinase A + mTORC1/2 inhibitor
Osimertinib+ Quaratusugene Ozeplasmid	NCT04486833	Phase I	TUSC2 TSG inhibitor
Osimertinib + MRX-2843	NCT04762199	Phase Ib	MERTK/FLT3 inhibitor
Osimertinib + Tegavivint	NCT04780568	Phase Ib	TBL1 inhibitor
BLU-945	NCT04862780 (SYMPHONY)	Phase I/II	4Gen EGFR against C797S
Lazertinib + Amivantamab	NCT04965090	Phase II	3rd gen EGFR + EGFR-MET bispecific Ab
Befotertinib + Icotinib	NCT05007938	Phase II	EGFR inhibitor
Osimertinib + LY3295668	NCT05017025	Phase Ib/II	Aurora Kinase A inhibitor
BLU-701	NCT05153408 (HARMONY)	Phase I/II	4Gen EGFR inhibitor against C797S
JIN-A02	NCT05394831	Phase I/II	4Gen EGFR inhibitor against C797X

Relatively few trials are underway that directly address MRD and the evasion of apoptosis. This may be owing to the novelty of the proposed mechanisms involved, as well as the paucity of literature demonstrating a clear role for apoptosis-targeting agents in lung cancer. To date, the phase II study of combination osimertinib with MEK1/2 inhibitor selumetinib (NCT03392246) is the only trial currently underway that is evaluating whether this combination may prevent the emergence of acquired resistance.

## Conclusions

EGFR-TKI therapy remains the mainstay of first-line treatment for *EGFR*-mutant NSCLC however this rarely leads to cure as acquired resistance invariably develops. The understanding of the MRD state that develops after initial exposure to EGFR-TKIs such as YAP/TEAD provide an insight into how tumour cells escape from initial apoptosis.

The pre-clinical success of osimertinib and XAV939 demonstrates a promising alternative of targeting multiple pathways proactively in the first line setting to deepen MRD that may circumvent the development of drug resistance and prove to be beneficial than targeting the acquired resistance pathways that develop post osimertinib exposure. Further trials are required to develop a more effective treatment strategy and evaluate the efficacy and safety of upfront combination or sequential targeted therapies. The ongoing discovery of more targetable resistance mechanisms are likely to continue to reshape the future treatment of *EGFR-*mutant NSCLC. However, a strategy which involves optimal EGFR inhibition and eliminating these MRD cells which have senescent properties, is potentially the key to unlocking the ‘holy grail’ of cure in this disease.

## Author contributions

Conceptualisation, SA and NB. Supervision, SA. Writing, review, and editing, NB, YW, and SA.

## Acknowledgments

SA would like to acknowledge the Royal Australasian College of Physicians for the Robert Maple-Brown Research Establishment Fellowship as post-doctoral fellowship support.

## Conflict of interest

SA Speaker fees: Merck-Sharpe & Dohme, Astra Zeneca, Roche, and Bristol-Myers Squibb. Travel support: Astra Zeneca, Roche, Merck-Sharpe & Dohme. Advisory boards: Boehringer Ingelheim, Roche. Non-financial aid: Astra Zeneca, Pfizer.

The remaining authors declare that the research was conducted in the absence of any commercial or financial relationships that could be construed as a potential conflict of interest.

## Publisher’s note

All claims expressed in this article are solely those of the authors and do not necessarily represent those of their affiliated organizations, or those of the publisher, the editors and the reviewers. Any product that may be evaluated in this article, or claim that may be made by its manufacturer, is not guaranteed or endorsed by the publisher.
